# Sevoflurane and isoflurane inhibit KCl-induced Class II phosphoinositide 3-kinase α subunit mediated vasoconstriction in rat aorta

**DOI:** 10.1186/s12871-016-0227-9

**Published:** 2016-08-18

**Authors:** Shaozhong Yang, Qi Wu, Shanshan Huang, Zi Wang, Feng Qi

**Affiliations:** Department of Anesthesiology, Qilu Hospital of Shandong University, Jinan, 250012 China

**Keywords:** Sevoflurane, Isoflurane, KCl, Phosphoinositide 3 kinase, Rho kinase, Myosin light chain phosphatase

## Abstract

**Background:**

Class II phosphoinositide 3-kinase α-isoform (PI3K-C2α) is involved in regulating KCl-induced vascular smooth muscle contraction. The current study was to investigate the effects of sevoflurane (SEVO) and isoflurane (ISO) on KCl-elicited PI3KC2α mediated vasoconstriction in rat aortic smooth muscle.

**Methods:**

Isometric force, in the absence or presence of SEVO or ISO (1 ~ 3 minimum alveolar concentration, MAC), PI3K inhibitor LY294002, Rho kinase inhibitor Y27632, and membrane translocation of PI3K-p85, PI3K-C2α, Rho kinase (Rock II), or phosphorylation of MYPT1/Thr853, MYPT1/Thr696, CPI-17/Thr38 and MLC in response to KCl (60 mM) was measured by using isometric force transducer and western blotting analysis, respectively.

**Results:**

KCl elicited a rapid and sustained contraction of rat aortic smooth muscle that was inhibited by both SEVO and ISO in a concentration-dependent manner, and also suppressed by LY294002 (1 mM) and Y27632 (1 uM). LY294002 (1 mM) and Y27632 (1 uM) also inhibited KCl-induced MLC phosphorylation. LY294002 (1 mM) inhibited KCl-induced PI3K-p85, PI3K-C2α membrane translocation in response to KCl (*p* <0.05, *p* < 0.01, respectively). Not only Y27632 (1 uM), but also LY294002 (1 mM), inhibited KCl-induced Rock-II membrane translocation (*p* < 0.01). SEVO and ISO inhibited KCl-stimulated MLC phosphorylation, PI3K-C2α and Rock-II,not PI3K p85 membrane translocation in a concentration-dependent manner in rat aorta. Both SEVO and ISO suppressed the MYPT1/Thr853, not MYPT1/Thr696 and CPI-17/Thr38, MLC phosphorylation in response to KCl.

**Conclusion:**

PI3K-C2α mediates part of SEVO and ISO-mediated vasodilation in rat aorta. The cellular mechanisms underlying the inhibitory effect of volatile anesthetics might be mediated by KCl/PI3K-C2α/Rho kinase/MYPT1/MLC pathway.

## Background

**Fig. 1:**
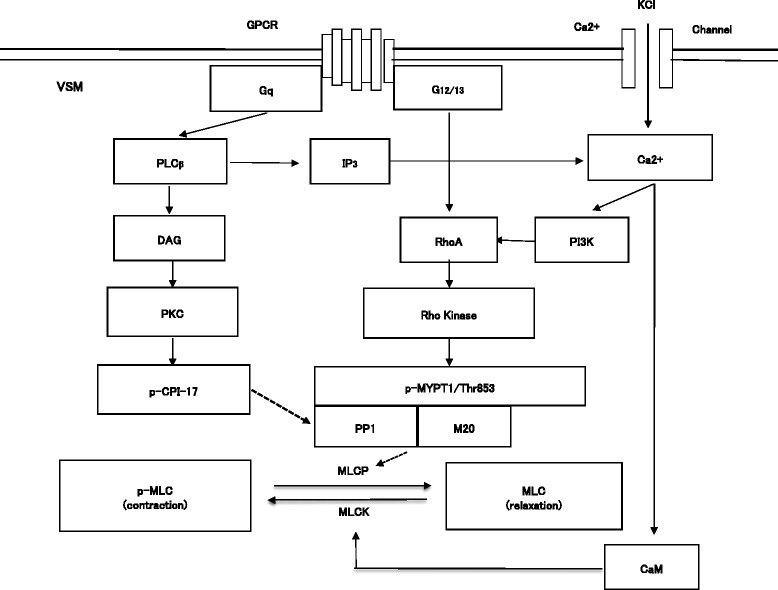
Schematic illustration of volatile anesthetics on Ca^2+^-induced Ca^2+^ sensitization. VSM = vascular smooth muscle, GPCR = G protein coupled receptor, G12/13 = 12/13 subunit of heterotrimeric G-protein; Gq = q subunit of heterotrimeric G-protein, PLC = phospholipase C, IP3 = inositol 1,4,5-triphosphate, DAG = diacylglycerol, PKC = protein kinase C, CPI-17 = PKC-potentiated inhibitory protein, KCl = potassium chloride, CaM = calmodulin, PI3K-C2α = Class II phosphoinositide 3 kinase α-isoform, Rho kinase = small GTPase ROCK II, MLC = myosin light chain, p-MLC = phosphorylated MLC, MLCK = MLC kinase, MLCP = MLC phosphatase, MYPT1/853 = myosin phosphatase target subunit Thr853, MYPT1/696 = myosin phosphatase target subunit Thr696, PP1 = catalytic subunit of Type 1 protein phosphatase, M20 = accessory 20 kDa subunit, Dotted line = inhibitory effect

The mechanism of KCl-stimulated vascular smooth muscle (VSM) contraction may involve Ca^2+^-dependent and Ca^2+^ sensitization pathways. Membrane depolarization by KCl increases Ca^2+^ influx via voltage-dependent Ca^2+^ channel receptors on VSM cells, leading to an increase in cytosolic free Ca^2+^ ([Ca^2+^]_i_). This increase in [Ca^2+^]_i_ activates Ca^2+^-calmodulin-dependent myosin light chain kinase, resulting in MLC phosphorylation and vascular contraction [1–4]. In addition to the Ca^2+^-dependent pathway, the Ca^2+^-induced Ca^2+^ sensitization pathway may also regulate MLC phosphatase (MLCP) through modulation of class II phosphoinositide 3 kinase α subunit (PI3K-C2α) and Rho kinase activity, which results in vascular contraction [5–8] (Fig. 1).

PI3Ks are a ubiquitously expressed enzyme family that phosphorylates membrane inositol lipids and exert diverse biological activities [9, 10]. PI3Ks are divided into three main classes. VSM is known to express multiple PI3Ks, including the class I enzymes p110α and p110β, and class II enzymes PI3K-C2α and C2β [11].

Several studies show that sevoflurane (SEVO) and isoflurane (ISO) can provide protection against ischemia-reperfusion injury via the PI3K/Akt pathway [12–15]. In contrast, volatile anesthetics can result in some complications, such as hypertension, particularly at higher concentrations. The mechanism may involve relaxation of VSM directly through intracellular signaling transduction pathways [1, 16–18]. However, the exact mechanism is not fully understood.

We previously demonstrated that SEVO inhibited GTPγ S-stimulated Rho-Rho kinase-mediated vasoconstriction in isolated rat aortic VSM [16]. We also revealed that SEVO attenuated Angiotensin II (Ang II)-induced vasoconstriction by reducing PKC phosphorylation without affecting [Ca^2+^]_i_ in VSM [17]. We recently demonstrated that SEVO inhibited MLC, the PKC-potentiated inhibitory protein CPI-17, and myosin phosphatase target subunit MYPT1/Thr853 phosphorylation in response to Ang II. ISO also inhibited MLC phosphorylation in response to Ang II, which was associated with a decrease in MYPT1/Thr853, but not in CPI-17 phosphorylation. Neither SEVO nor ISO affected Ang II-induced phosphorylation of MYPT1/Thr696 [18]. However, little information is available about the effect of SEVO and ISO on Ca^2+^-induced Ca^2+^ sensitization in regulating VSM contraction. The current study involved investigating the effects of volatile anesthetic modulation of PI3K subunit and Rho kinase activity on vasoconstriction in response to KCl in rat isolated aortic smooth muscle.

## Methods

### Vascular smooth muscle tissue preparation

This protocol was approved by Shandong University Animal Care and Use Committee and Qilu Hospital of Shandong University Medical Ethics Committee (No. KYLL-2013-51). Aortic rings and strips were prepared according to previous protocols [16–18]. Male Wistar rats (250–350 g) were anesthetized with halothane and euthanized by exsanguination from the common carotid artery. The descending thoracic aorta was carefully dissected, and adherent fat and connecting tissue were removed. The endothelium was removed by gentle rubbing of the internal surface with a stainless steel needle.

### Isometric force measurement

**Fig. 2:**
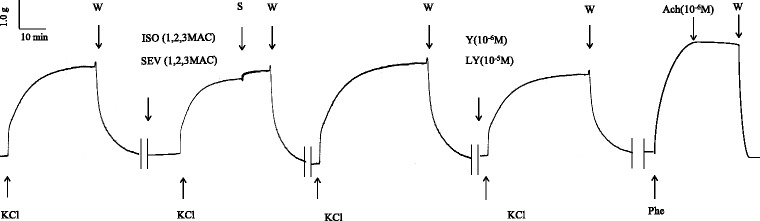
The protocol of sevoflurane, isoflurane, LY294002 and Y27632 on KCl-induced contraction of rat aortic smooth muscle. X axis = Time (min), Y axis = Tension (g), W = wash, S = stop, ISO = isoflurane, SEVO = sevoflurane, MAC = minimum alveolar concentration, Y = Y27632, LY = LY294002, Phe = phenylephedrine, Ach = acetylcholine

In this study, KCl (60 mM) was used as an agonist according to previous results [7]. KCl-elicited tension was measured using isometric force methods as described previously [16–18]. Briefly, endothelium-denuded aortic rings were equilibrated under a resting tension of 3 g in Krebs bicarbonate solution (KBS) (in mmol/L, NaCl, 118.2; KCl, 4.6; CaCl_2_, 2.5; KH_2_PO_4_, 1.2; MgSO_4_, 1.2; NaHCO_3_, 24.8; and dextrose 10) at 37 °C and gassed with a mixture of 95 % (v/v) O_2_ and 5 % (v/v) CO_2_ with a fresh gas flow of 2 L/min. After 60 min equilibration, with the bathing fluid replaced every 20 min, the aortic rings were incubated with KCl (60 mM) to access their overall contractile responsiveness. Removal of the endothelium was confirmed with 3 × 10^−7^ M phenylephrine pre-contracted vessels that showed a lack of relaxation in the presence of 10^−5^ M acetylcholine. To examine the effect of anesthetics on KCl-induced contraction, six aortic rings from each individual rat (*n* = 7) were randomly exposed to 0, 1, 2, and 3 minimum alveolar concentration (MAC) of each anesthetic, 1 mM LY294002 (PI3K inhibitor), or 1 μM Y27632 (Rho kinase inhibitor) for 15 min before the addition of KCl (Fig. 2). Isometric force development in response to KCl was expressed as a percentage relative to that induced by KCl.

SEVO or ISO was delivered into the gas mixture with a fresh gas flow of 2 L/min via a calibrated agent-specific vaporizer (Penlon, Abingdon, UK) to aerate the KBS with equivalent human MAC. The concentration of the resulting gas mixture was monitored and adjusted using an Atom 303 anesthetic agent monitor (Atom, Tokyo, Japan). Our previous data using the same experimental system indicated that the concentrations of SEVO or ISO in KBS measured by gas chromatography (Shimazu Seisakusho, Tokyo, Japan) were 0.17, 0.28, and 0.41 mM for gas concentrations of 1.7 % (1 MAC), 3.4 % (2 MAC), and 5.1 % (3 MAC) SEVO, respectively (*n* = 8–12) and 0.19, 0.39, and 0.56 mM for gas concentrations of 1.2 % (1 MAC), 2.3 % (2 MAC), and 3.5 % (3 MAC) ISO, respectively (*n* = 8–12), after 15 min of equilibration.

### Phosphorylation of MYPT1, CPI-17, and MLC

For measurement of MYPT1, CPI-17, and MLC phosphorylation, the endothelium-denuded strips (approximately 3.5 cm in length) were bathed in oxygenated KBS and were equilibrated for 60 min before the start of the experiment. One strip was obtained from each animal.

Previous data demonstrated that KCl (60 mM) induced a rapid increase in MLC phosphorylation and reached peak levels approximately 5 min after exposure to KCl [8]. Therefore, the later experiments were examined 5 min after the application of KCl. Aortic strips from different animals were incubated with 0 (control), 60 mM KCl, 1 MAC SEVO, 2 MAC SEVO, 1 mM LY294002, and 1 μM Y27632, or 0 (control), 60 mM KCl, 1 MAC ISO or 2 MAC ISO, 1 mM LY294002, and 1 μM Y27632 for 15 min without KCl or 5 min in the presence of KCl, and were quickly frozen with liquid nitrogen. Thereafter, protein phosphorylation was measured using western blotting analysis as described previously [10–12]. Briefly, the frozen strips were cut into small pieces and homogenized in ice-cold lysis buffer with 0.2 % (v/v) Triton X-100. Homogenates were centrifuged at 10,000 g for 30 min at 4 °C. The supernatants were assayed for protein concentration using the bicinchoninic acid method [13] and subsequently used for detection of MYPT1, CPI-17, and MLC phosphorylation.

In each experiment, proteins (30 μg) were separated by sodium dodecyl sulfate-polyacrylamide gel electrophoresis (12 % or 5 %) and transferred to nitrocellulose membranes. Membranes were incubated with anti-MLC, anti-CPI-17, anti-MYPT1/Thr696, anti-MYPT1/Thr853 (1:1000, 1:1000, 1:500, and 1:500, respectively), and antiphospho-MLC, antiphospho-CPI-17, antiphospho-MYPT1/Thr696, and antiphospho-MYPT1/Thr853 antibodies (1:1000, 1:1000, 1:500, and 1:500, respectively) for 2 h, followed by incubation with a horseradish peroxidase-conjugated antibody (1:2000) for 1 h [12].

The densities of immunoreactive bands were detected using chemiluminescence (Amersham Pharmacia Biotech, Piscataway, NJ, USA) and were assessed with image analysis software (NIH Image 1.62; National Institute of Health, Bethesda, MD, USA). Bands with molecular weights of 17 kDa, 20 kDa, and 110 kDa were identified as CPI-17, MLC, and MYPT1, respectively. The ratios of phosphorylated to total CPI-17, MLC, and MYPT1 were used as an indicator of activation of each enzyme and expressed as the percentage relative to the baseline control level (referred to as 100 %).

### Membrane translocation of PI3K and Rho kinase (Rock II)

For enzyme membrane translocation, PI3K and Rho kinase (Rock II) activity were measured using western blotting analysis as described previously [16]. Briefly, the endothelium-denuded aortic strips were bathed in 20-mL organ chambers containing KBS solution. The strips were equilibrated for 60 min in control KBS, which was replaced every 20 min. To measure the dose effect of SEVO, ISO, and kinase inhibition on KCl-stimulated membrane translocation of PI3K or Rock II, some aortas were treated with either 1.7 % SEVO, 3.5 % SEVO, 1.2 % ISO, 2.3 % ISO, 1 mM LY294002, or 1 μM Y27632 for 15 min before exposure to KCl. After treatment with KCl for 5 min, the samples were then rapidly frozen with liquid nitrogen.

Frozen aortas were cut into small pieces and were homogenized in ice-cold lysis buffer containing 1 mM Tris/HCl, pH 7.5, 5.5 mM MgCl_2_, 100 mM NaCl, 2 mM EDTA, 1 mM 4-(2-aminoethyl) benzonesulfonyl fluoride, 20 μg/mL leupeptin, 20 μg/mL aprotinin, and 1 mM Na_3_VO_4_. Homogenates were centrifuged at 13,000 g for 15 min at 4 °C, and the supernatants were collected and centrifuged at 100,000 g for 30 min at 4 °C again. The supernatant (cytosolic fraction) was removed, and the pellet (membrane fraction) was re-suspended using the same lysis buffer. The protein concentrations of each fraction were determined using the bicinchoninic acid method [19].

Equal amounts of total protein (30 μg) were used for every sample in each experiment. Proteins were separated by 7.5 % or 12 % sodium dodecyl sulfate-polyacrylamide gel electrophoresis and were transferred to a nitrocellulose membrane. The membrane was treated with anti-PI3K-p85α, anti-PI3K-C2α, and anti-Rock-II antibodies (1:200, 1:200, and 1:500, respectively) for 4 h, followed by incubation with a horseradish peroxidase-conjugated antibody (1:2000) for 1 h. Immunoreactive bands were detected using chemiluminescence (Amersham Pharmacia Biotech, Piscataway, NJ, USA) and were assessed with image analysis software (NIH image 1.62; National Institute of Health). The amount of PI3K-p85α, PI3K-C2α, and Rock-II on the membrane was expressed as a percentage of the total value (i.e., membrane fraction plus cytosolic fraction).

### Materials and reagents

SEVO and ISO were purchased from Dainabot Company Limited (Osaka, Japan). Polyclonal antibodies against phospho-MLC (Thr18/Ser19), phospho-MYPT1 (Thr853), phospho-MYPT1 (Thr696), phospho-CPI-17 (Thr38), MLC2 (FL-172), MYPT1 (H-130), CPI-17 (H60), PI3K-p85α, and Rock-II, and the secondary antibody labeled with horseradish peroxidase (HRP) were all obtained from Santa Cruz Biotechnology, Inc. (Santa Cruz, CA, USA). PI3K-C2α was purchased from Abcam, Inc. (Tokyo, Japan). LY294002 was provided from Merck-Calbiochem Biosciences (Darmstadt, Germany). Y27632 was purchased from Calbiochem-Novabiochem Corporation (San Diego, CA, USA). All other reagents for experiments and western blotting were of analytical grade.

### Statistical analysis

Data analyses were performed using the software program StatMate (Atoms, Tokyo, Japan). The sample size (*n*) refers to the number of rats from which the aortas were harvested. Data are presented as the mean ± standard deviation and were analyzed using the Dunnett test after analysis of variance for multiple comparison of isometric tension or protein phosphorylation. A *p*-value < 0.05 was considered significant.

## Results

**Fig. 3:**
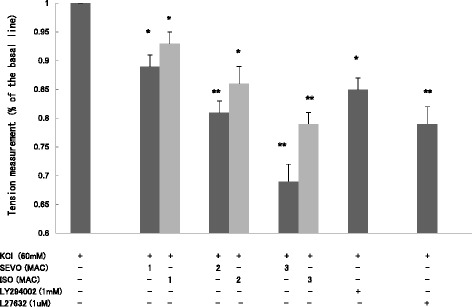
Effects of sevoflurane and isoflurane on KCl-induced contraction in rat aortic smooth muscle. The aortas rings were incubated in the presence of 1.7 %, 3.4 %, 5.1 % sevoflurane (SEVO) or 1.2 %, 2.3 %, 3.5 % isoflurane (ISO), or 1 mM LY294002, or 1 uM Y27632 for 15 min before and during exposure to KCl. Isometric tension development in response to anesthetic or inhibitor was expressed as percentage relative to that induced by KCl. * *P* < 0.05, ** *P* < 0.01 versus the value in the presence of KCl without anesthetics or inhibitor (*n* = 7, each)

KCl induced rapid contraction and reached a maximum level approximately 5 min after, which was sustained for more than 30 min in rat aortic rings (Fig. 2). SEVO and ISO attenuated KCl-induced contraction in a concentration-dependent manner, and the contraction was also suppressed by LY294002 (1 mM) and Y27632 (1 μM). There was no statistical difference between SEVO and ISO when comparing equipotent concentrations (Fig. 3).

**Fig. 4:**
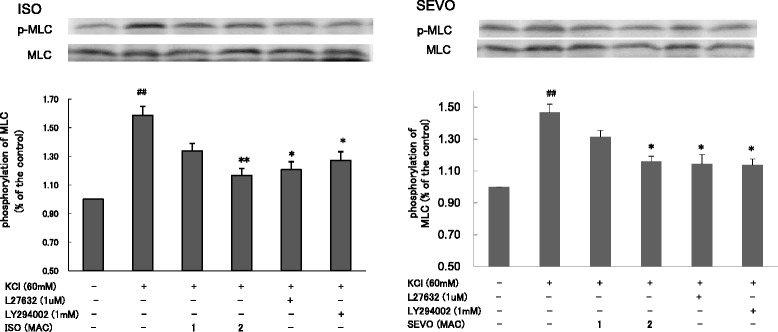
Effects of sevoflurane and isoflurane on KCl-induced MLC phosphorylation in rat aortic smooth muscle. The aortas strips were incubated in the presence of 1.7 %, 3.4 % sevoflurane (SEVO) or 1.2 %, 2.3 % isoflurane (ISO), or 1 mM LY294002, or 1 uM Y27632 for 15 min before and during exposure to KCl. MLC phosphorylation development in response to anesthetic or inhibitor was expressed as percentage relative to that induced by KCl. * *P* < 0.05, ** *P* < 0.01 versus the value in the presence of KCl without anesthetics or inhibitor, ## *P* < 0.01 compared with the baseline without KCl (*n* = 7, each)

**Fig. 5:**
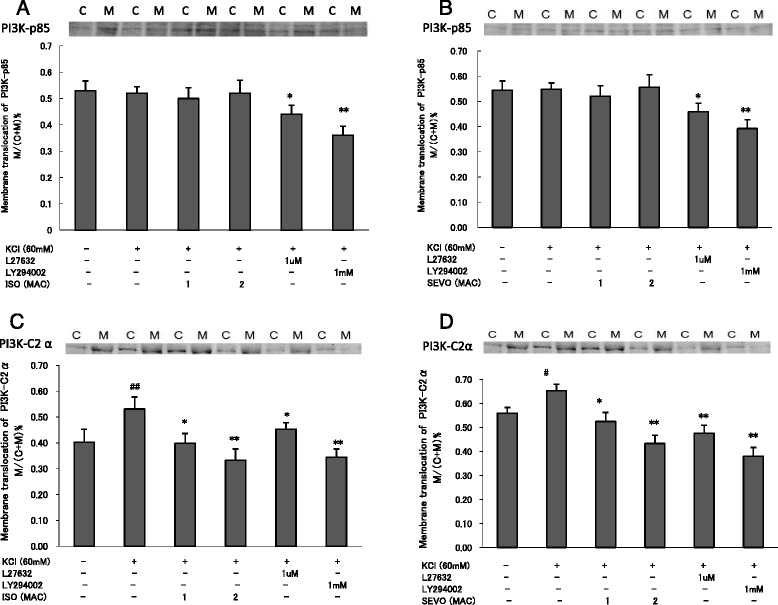
Effects of sevoflurane and isoflurane on member translocation of PI3K-C2α and PI3K-p85 in response to KCl. The aortic strips were incubated in the presence of 0, 1.7 %, 3.4 % sevoflurane (SEVO), or 0, 1.2 %, 2.3 % isoflurane (ISO), or 10 uM, 1 mM LY294002 for 15 min before and during exposure to KCl. The amount of PI3K-p85α (A and B) and PI3K-C2α (C and D) on the membrane was expressed as a percentage of the total value (i.e., membrane fraction plus cytosolic fraction). * *P* < 0.05, ** *P* < 0.01 versus the value in the presence of KCl, ## *P* < 0.01 compared with the baseline without KCl (*n* = 7, each)

**Fig. 6:**
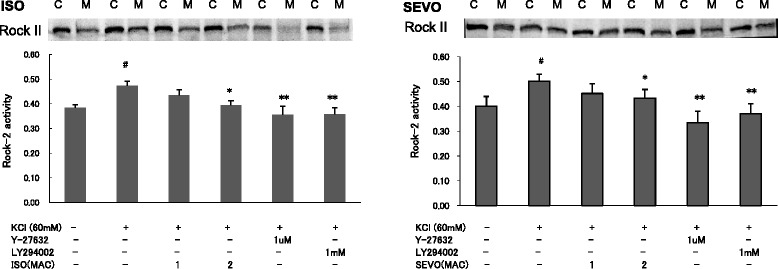
Effects of sevoflurane and isoflurane on member translocation of Rho kinase (Rock II) in response to KCl. The aortic strips were incubated in the presence of 0, 1.2 %, 2.3 % isoflurane (ISO,A), or 0, 1.7 %, 3.4 % sevoflurane (SEVO,B), or 1 mM LY294002, or 1 uM Y27632 for 15 min before and during exposure to KCl. The amount of Rock-II on the membrane was expressed as a percentage of the total value (i.e., membrane fraction plus cytosolic fraction). * *P* < 0.05, ** *P* < 0.01 versus the value in the presence of KCl, # *P* < 0.05 compared with the baseline without KCl (*n* = 7, each)

Consistent with the change in isometric force measurements, SEVO and ISO also inhibited MLC phosphorylation in a concentration-dependent manner in response to KCl (Fig. 4). SEVO and ISO inhibited membrane translocation of PI3K-C2α (Fig. 5c and d) and Rock-II (Fig. 6), but not PI3K-p85 (Fig. 5a and b) in response to KCl in a concentration-dependent manner in rat aortas.

**Fig. 7:**
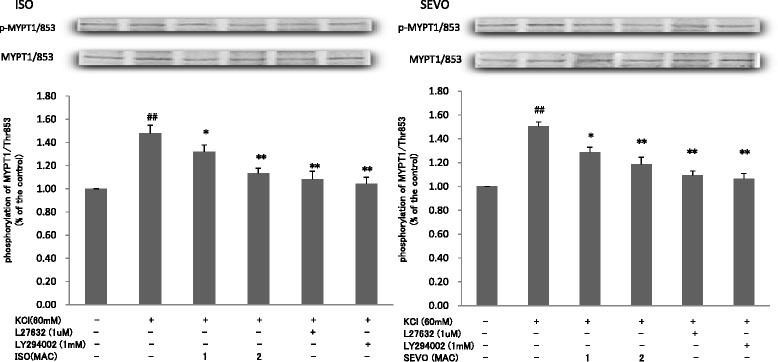
Effects of sevoflurane and isoflurane on KCl-induced MYPT1/853 phosphorylation in rat aortic smooth muscle. The aortas strips were incubated in the presence of 1.7 %, 3.4 % sevoflurane (SEVO) or 1.2 %, 2.3 % isoflurane (ISO), or 1 mM LY294002, or 1 uM Y27632 for 15 min before and during exposure to KCl. MYPT1/853 phosphorylation development in response to anesthetic or inhibitor was expressed as percentage relative to that induced by KCl. * *P* < 0.05, ** *P* < 0.01 versus the value in the presence of KCl without anesthetic or inhibitor, ## *P* < 0.01 compared with the baseline without KCl (*n* = 7, each)

**Fig. 8:**
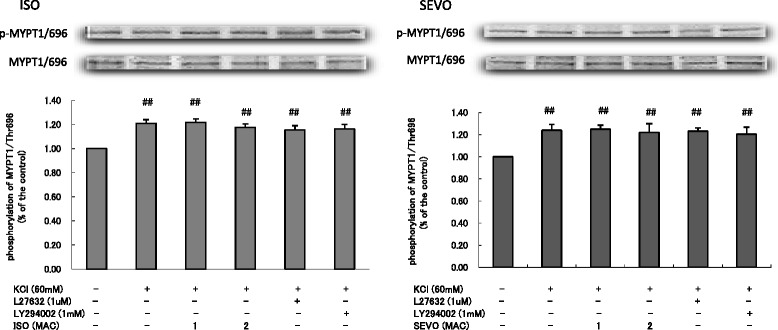
Effects of sevoflurane and isoflurane on KCl-induced MYPT1/696 phosphorylation in rat aortic smooth muscle. The aortas strips were incubated in the presence of 1.7 %, 3.4 % sevoflurane (SEVO) or 1.2 %, 2.3 % isoflurane (ISO), or 1 mM LY294002, or 1 uM Y27632 for 15 min before and during exposure to KCl. MYPT1/696 phosphorylation development in response to anesthetic or inhibitor was expressed as percentage relative to that induced by KCl. ## *P* < 0.01 compared with the baseline without KCl (*n* = 7, each)

**Fig. 9:**
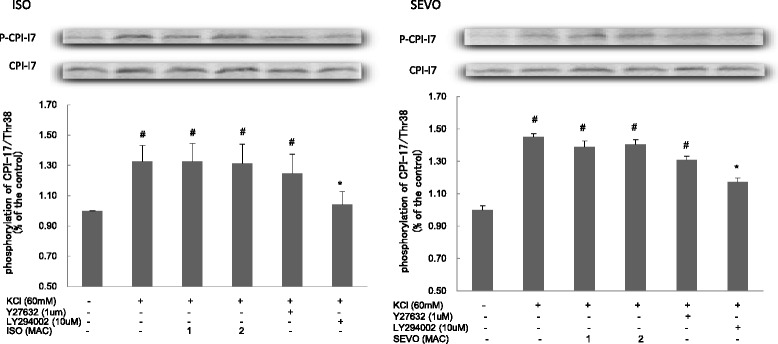
Effects of sevoflurane and isoflurane on KCl-induced CPI-17 phosphorylation in rat aortic smooth muscle. The aortas strips were incubated in the presence of 1.7 %, 3.4 % sevoflurane (SEVO) or 1.2 %, 2.3 % isoflurane (ISO), or 1 mM LY294002, or 1 uM Y27632 for 15 min before and during exposure to KCl. CPI-17 phosphorylation development in response to anesthetic or inhibitor was expressed as percentage relative to that induced by KCl. * *P* < 0.05 versus the value in the presence of KCl, # *P* < 0.05 versus the baseline without KCl (*n* = 7, each)

Both SEVO and ISO inhibited the phosphorylation MYPT1/Thr853 in response to KCl in a concentration-dependent manner (Fig. 7). Neither SEVO nor ISO affected the increase in the phosphorylation of MYPT1/Thr696 (Fig. 8) and CPI-17/Thr38 (Fig. 9) in response to KCl.

LY294002 (1 mM) inhibited KCl-induced PI3K-p85 and PI3K-C2α membrane translocation in response to KCl (*p* < 0.05, p < 0.01, respectively) (Fig. 5). In addition to Y27632 (1 μM), LY294002 (1 mM) also inhibited KCl-induced MLC phosphorylation (Fig. 4) and Rock-II membrane translocation in rat aortic smooth muscle (*p* < 0.01) (Fig. 6).

## Discussion and Conclusions

The current study demonstrated that both SEVO and ISO inhibit KCl-induced MLC phosphorylation and vasoconstriction by modulating PI3K-C2α and Rho kinase activity in the rat aorta. These findings suggest that the inhibition of Ca^2+^-induced Ca^2+^ sensitization induced by volatile anesthetics contributes to its vasodilatory effect.

Several studies demonstrate that LY294002 and Y27632 inhibit KCl-induced vasoconstriction by suppressing MYPT1 and CPI-17 phosphorylation in rat or rabbit aortic smooth muscle [8, 11]. LY294002 is similar to the broad-spectrum PI3K inhibitor wortmannin, which inhibits class I PI3Ks (α, β, γ, and δ), class II PI3Ks, and class III PI3Ks [20]. Previous studies have shown that the PI3K-C2α isoform is less sensitive to PI3K inhibitors compared with other PI3K isoforms [21, 22]. Therefore, we selected high concentrations of LY294002 to inhibit PI3K-C2α and MLC phosphorylation. In this study, our results showed that LY294002 and Y27632 inhibited KCl-induced vasoconstriction by suppressing MLC phosphorylation and contraction in rat aortic smooth muscle. We also demonstrated that LY294002 not only inhibits class II PI3K-C2α, but also class I PI3K-p85, as well as Rho kinase in response to KCl, suggesting that PI3K and/or Rho kinase may mediate the inhibition of KCl-induced Ca^2+^ sensitivity. Because specific PI3K-C2α-inhibitors do not exist, it remains unclear whether the reduced MLC phosphorylation and vasoconstriction depends on class II PI3K-C2α-inhibition or inhibition of class I PI3K.

Accumulating evidence demonstrates that PI3K-C2α and Rho kinase, or PKC, MYPT1/Thr853, and CPI-17 are involved in vasoconstriction by regulation of Ca^2+^-induced Ca^2+^ sensitization [8, 11, 23–25]. Previous data indicated that PKC is a major upstream activator of CPI-17 [26, 27]. Takuwa et al. reported that KCl membrane depolarization-induced CPI-17 phosphorylation was not inhibited by the protein kinase C inhibitor GF109203X [25]. It seems likely that PKC was not involved in modulating KCl-induced CPI-17 phosphorylation. Rho kinase can regulate MLCP activity by influencing MYPT1 phosphorylation at Thr853 and/or Thr696 [20]. It is possible that the inhibitory effect of SEVO and ISO on KCl-induced vasoconstriction was mainly mediated through the Ca^2+^/PI3K-C2α/Rho kinase/MYPT1/Thr853 pathway.

Several reports also showed that SEVO decreased myofilament Ca^2+^ sensitivity by regulating Rho kinase and PKC activity [1, 17, 18]. We recently showed that SEVO does not alter norepinephrine-induced intracellular Ca^2+^ changes in the diabetic rat aorta [28]. These data further demonstrate that myofilament Ca^2+^ sensitization, not Ca^2+^-dependent mechanisms, is involved in modulating vascular tension by SEV.

Evidence also demonstrates that ISO inhibition of agonist-induced contraction of Ca^2+^ sensitivity mechanisms is mainly mediated by increasing Ca^2+^ influx via voltage-dependent calcium channels [29, 30]. We previously demonstrated that ISO exhibited no inhibitory effect on PKC phosphorylation in response to Ang II up to a concentration of 3.5 % [31]. These data indicate that ISO-regulated vasoconstriction is mainly through the Ca^2+^-dependent pathway and not through the Ca^2+^ sensitivity pathway. In contrast to the above results, we recently demonstrated that ISO inhibits Ang II-induced vasoconstriction through regulating the phosphorylation of the MLCP subunit MYPT1/Thr853 [18]. Furthermore, we demonstrated that ISO inhibits KCl-elicited MLC phosphorylation and vasoconstriction by suppressing MYPT1/Thr853, and not CPI-17 phosphorylation in the rat aorta. These data show that the Ca^2+^-induced Ca^2+^ sensitization pathway was also involved in the regulation of vasoconstriction by ISO.

Overall, our data, in combination with other investigations, reveal that inhibition of KCl-elicited vasoconstriction by volatile anesthetics may involve the modulation of PI3K-C2α and/or Rho-Rho kinase activity, the upstream activators of MLCP [8, 11, 25]. Consistent with these data, the current investigation confirmed that both SEVO and ISO suppress the vasoconstriction of Ca^2+^-induced Ca^2+^ sensitization through modulating PI3K-C2α, Rho kinase activity, and MYPT1/Thr853 phosphorylation in rat vascular smooth muscle.

However, this study has some limitations: (1) the aim of this study was to elucidate the cellular mechanisms responsible for anesthetic-induced inhibition of vasoconstriction. Therefore, we used endothelium-denuded aortic strips or rings for this study. Indeed, the endothelium plays an important role in regulating systemic vascular tone under *in vivo* conditions. However, we used endothelium-denuded samples to avoid the effects of the endothelium. (2) Moreover, pre-arteriolar resistance arteries, not conduit arteries, mainly determine peripheral vascular resistance. Therefore, the current results obtained from endothelium-denuded aortas cannot be used to directly extrapolate to the *in vivo* condition. (3) Furthermore, the aortic rings are not necessarily reflective of the physiology of resistance arteries and thus may not be ideally suited to address issues related to the clinical hemodynamic effects of volatile anesthetics.

In conclusion, our study demonstrates that both SEVO and ISO inhibit KCl-induced PI3K-C2α-participation, Rho kinase-mediated MLC phosphorylation, and vasoconstriction in rat aortic smooth muscle. The intracellular mechanisms of regulating VSM contraction of volatile anesthetics may be mediated by the KCl/PI3K-C2α/Rho kinase/MLCP/MLC pathway.

## Abbreviations

Ang II, Angiotensin II; BCA, bicinchoninic acid; CaM, Ca2 + -calmodulin; CPI-17/Thr38, PKC-potentiated inhibitory protein/Thr38; GPCR, G protein-coupled receptors; ISO, isoflurane; MAC, minimum alveolar concentration; MLC, myosin light chain; MLCK, myosin light chain kinase; MLCP, MLC phosphatase; MYPT1/Thr696, myosin phosphatase target subunit/Thr696; MYPT1/Thr853, myosin phosphatase target subunit/Thr853; PI3K-C2α, Class II phosphoinositide 3-kinase α-isoform; SDS-PAGE, sodium dodecyl sulfate-polyacrylamide gel electrophoresis; SEVO, sevoflurane; VSM, vascular smooth muscle

## References

[1] Akata T (2007). General anesthetics and vascular smooth muscle. Anesthesiology..

[2] Ratz PH, Berg KM, Urban NH, Miner AS (2005). Regulation of smooth muscle calcium sensitivity: KCl as a calcium-sensitizing stimulus. Am J Physiol Cell Physiol..

[3] Ito T, Ikebe M, Kargacin GJ, Hartshorne DJ, Kemp BE, Fay FS (1989). Effects of modulators of Myosin light-chain kinase activity in single smooth muscle cells. Nature..

[4] Somlyo AP, Somlyo AV (1994). Signal transduction and regulation in smooth muscle. Nature..

[5] Dimopoulos GJ, Semba S, Kitazawa K, Eto M, Kitazawa T (2007). Ca2 + -dependent rapid Ca2+ sensitization of contraction in arterial smooth muscle. Circ Res..

[6] Mita M, Yanagihara H, HoshinumaI S, Saito M, Walsh MP (2002). Membrane depolarization-induced contraction of rat caudal arterial smooth muscle involves Rho-associated kinase. Biochem J..

[7] Sakurada S, Takuwa N, Sugimoto N, Wang Y, Seto M, Sasaki Y, Takuwa Y (2003). Ca2 + -dependent activation of Rho and Rho kinase in membrane depolarization-induced and receptor stimulation-induced vascular smooth muscle contraction. Circ Res..

[8] Yoshioka K, Sugimoto N, Takuwa N, Takuwa Y (2007). Essential role for class II phosphoinositide 3-kinase alpha-isoform in Ca2+-induced, Rho- and Rho kinase-dependent regulation of myosin phosphatase and contraction in isolated vascular smooth muscle cells. Mol Pharmacol.

[9] Foster FM, Traer CJ, Abraham SM, Fty MJ (2003). The phosphoinositide (PI) 3-kinase family. J Cell Sci..

[10] Rameh LE, Cantley LC (1999). The role of phosphoinositide 3-kinase lipid products in cell function. J Biol Chem..

[11] Wang Y, Yoshioga K, Azam MA, Takuwa N, Sakurada S, Kayaba Y, Sugimoto N, Inoki I, Kimura T, Kuwaki T, Takuwa Y (2006). Class II phosphoinositide 3-kinase α-isoform regulates Rho, myosin phosphatase and contraction in vascular smooth muscle. Biochem J..

[12] Raphael J, Rivo J, Gozal Y (2005). Isoflurane-induced myocardial preconditioning is dependent on phosphatidylinositol-3-kinase/Akt signaling. Br J Anesth..

[13] Chiari PC, Bienengraeber MW, Pagel PS, Krolikowski JG, Kersten JR, Warltier DC (2005). Isoflurane protects against myocardial infarction during early reperfusion by activation of phosphatidylinositol-3-kinase signal transduction: evidence for anesthetic-induced postconditioning in rabbits. Anesthesiology..

[14] Li H, Wang JK, Zeng YM, Yang CX, Chen HT, Wen XJ, Shui CL, Liang H (2008). Sevoflurane post-conditioning protects against myocardial reperfusion injury by activation of phosphatidylinositol-3-kinase signal transduction. Clin Exp Pharmacol Physiol..

[15] Wang JK, Yu LN, Zhang FJ, Yang MJ, Yu J, Yan M, Chen G (2010). Postconditioning with sevoflurane protects against focal cerebral ischemia and reperfusion injury via PI3K/Akt pathway. Brain Res..

[16] Yu J, Ogawa K, Tokinaga Y, Hatano Y (2003). Sevoflurane inhibits guanosine 5’-[γ-thio] triphosphate-stimulated, Rho/Rho kinase-mediated contraction of rat aortic smooth muscle. Anesthesiology..

[17] Yu J, Tokinaga Y, Ogawa K, Iwahashi S, Hatano Y (2004). Sevoflurane inhibits angiotensin II-induced, protein kinase C-mediated but not Ca2 + -elicited contraction of rat aortic smooth muscle. Anesthesiology..

[18] Qi F, Ogawa K, Tokinaga Y, Uematsu N, Minonishi T, Hatano Y (2009). Volatile anesthetics inhibit angiotensin II-induced vascular contraction by modulating myosin light chain phosphatase inhibiting protein, CPI-17 and regulatory subunit, MYPT1 phosphorylation. Anesth Analg..

[19] Smith PK, Krohn RI, Hermanson GT, Mallia AK, Gartner FH, Provenzano MD, Fujimoto EK, Goeke NM, Olson BJ, Klenk DC (1985). Measurement of protein using bicinchoninic acid. Anal Biochem..

[20] Stein RC, Waterfield MD (2000). PI3-kinase inhibition: a target for drug development?. Mol Med Today..

[21] Domin J, Pages F, Volinia S, Rittenhouse SE, Zvelebil MJ, Stein RC, Waterfield MD (1997). Cloning of a human phosphoinositide 3-kinase with a C2 domain that displays reduced sensitivity to the inhibitor wortmannin. Biochem J..

[22] Kong D, Yamori T (2008). Phosphatidylinositol 3-kinase inhibitors: promising drug candidates for cancer therapy. Cancer Sci..

[23] Hirano K (2007). Current topics in the regulatory mechanism underlying the Ca2+ sensitization of the contractile apparatus in vascular smooth muscle. J Pharmacol Sci..

[24] Feng J, Ito M, Ichikawa K, Isaka N, Nishikawa M, Hartshorne DJ, Nakano T (1999). Inhibitory phosphorylation site for Rho-associated kinase on smooth muscle myosin phosphatase. J Biol Chem..

[25] Azam M, Yoshioka K, Ohkura S, Takuwa N, Sugimoto N, Sato K, Takuwa Y (2007). Ca2 + -independent, inhibitory effects of cyclic adenosine 5’-monophosphate on Ca2+ regulation of phosphoinositide 3-kinase C2α, Rho, and myosin phosphatase in vascular smooth muscle. J Pharm and Experi Ther..

[26] Woodsome TP, Eto M, Everett A, Brautigan DL, Kitazawa T (2001). Expression of CPI-17 and myosin phosphatase correlates with Ca2+ sensitivity of protein kinase C-induced contraction in rabbit smooth muscle. J Physiol..

[27] Eto M, Kitazawa T, Matsuzawa F, Aikawa S, Kirkbride J, Isozumi N, Nishimura Y, Brautigan D, Ohki S (2007). Phosphorylation-induced conformational switching of CPI-17 produces a potent myosin phosphatase inhibitor. Structure..

[28] Fujii K, Ogawa K, Tokinaga Y, Iranami H, Hatano Y (2010). Sevoflurane does not alter norepinephrine- induced intracellular Ca2+ changes in the diabetes rat aorta. Can J Anesth..

[29] Akata T, Kanna T, Yoshino J, Takahashi S (2003). Mechanisms of direct inhibitory action of isoflurane on vascular smooth muscle of mesenteric resistance arteries. Anesthesiology..

[30] Tsuchida H, Namba H, Takahashi S, Yamakage M, Fujita S, Notsuki E, Namki A (1993). Effects of halothane and isoflurane on cytosolic calcium ion concentration an contraction in vascular smooth muscle of rat aorta. Anesthesiology..

[31] Ishikawa A, Ogawa K, Tokinaga Y, Uematsu N, Mizumoto K, Hatano Y (2007). The mechanism behind the inhibitory effect of isoflurane on angiotensin II induced vascular contraction is different from that of sevoflurane. Anesth Analg.

